# Unwrapping the Global Financing Facility: understanding implications for women’s children’s and adolescent’s health through layered policy analysis

**DOI:** 10.1080/16549716.2025.2476820

**Published:** 2025-05-16

**Authors:** Mary V. Kinney, Doris Kwesiga, Joy E. Lawn, Ulla Walmisley, Meghan Bruce Kumar, Joël Arthur Kiendrébéogo, Phillip Wanduru, Peter Waiswa, Donat Shamba, Jitihada Baraka, Andes Chivangue, Georgina Msemo, Rosie Steege, Asha Sara George, Countdown GFF policy analysis collaboration

**Affiliations:** a School of Public Health, University of the Western Cape, Bellville, South Africa; b Department of Surgery, University of Cape Town, Cape Town, South Africa; c Data Synergy and Evaluations, African Population and Health Research Center, Nairobi, Kenya; d Department of Infectious Disease Epidemiology and International Health, London School of Hygiene & Tropical Medicine, London, UK; e Department of Nursing, Midwifery and Health, Northumbria University, Newcastle upon Tyne, UK; f Health Systems and Research Ethics, KEMRI-Wellcome Trust Programme, Nairobi, Kenya; g Department of Research, Expertise and Capacity Building, Recherche Pour la Santé et le Développement (RESADE), Ouagadougou, Burkina Faso; h Department of Public Health, University Joseph Ki-Zerbo, Ouagadougou, Burkina Faso; i Heidelberg Institute of Global Health, Medical Faculty and University Hospital, Heidelberg University, Heidelberg, Germany; j Department of Public Health, Institute of Tropical Medicine, Antwerp, Belgium; k School of Public Health, Makerere University College of Health Sciences, Kampala, Uganda; l lDepartment of Global Public Health, Karolinska Institutet, Stockholm, Sweden; m Department of Health Systems, Impact Evaluation and Policy, Ifakara Health Institute, Dar es Salaam, Tanzania; n N’weti, Maputo, Mozambique; o Global Financing Facility, Dar es Salaam, Tanzania; p Department of International Public Health, Liverpool School of Tropical Medicine, Liverpool, UK; q South African Medical Research Council, South Africa

**Keywords:** Global Financing Facility for Women, Children and Adolescents: Examining National Priorities, Processes and Investments, Global health initiative, Global Financing Facility, health financing, external financing, policy analysis

## Abstract

The Global Financing Facility (GFF), launched in 2015, aims to catalyse funding for reproductive, maternal, newborn, child, and adolescent health, and nutrition. Few independent assessments have evaluated its processes and impact. We conducted a multi-layered policy analysis of GFF documents – the Investment Cases (ICs) and the GFF-linked World Bank Project Appraisal Documents (PADs) – examining the content of GFF documents for 28 countries, comparing four tracer themes (maternal and newborn health, adolescent health, community health, and quality), and analysing the policy processes in four country studies (Burkina Faso, Mozambique, Tanzania, and Uganda). From 2015 to 2022, GFF-linked PADs reported US$ 14.5 billion of funding across 26 countries through 30 PADs, with GFF contributing 4% to this value. GFF investments primarily focused on service delivery, governance, and performance-based financing. Countries received more targeted investments for maternal and newborn health and adolescent health linked to their burden of these tracer themes. Attention to community health and quality varied. ICs were broader than PADs and more inclusive in their development. Local contexts shaped policy processes. GFF supported priority-setting and learning; however, translating priorities into resourced actions proved challenging. Power dynamics influenced country ownership, donor coordination and resource mobilisation. The GFF is a significant opportunity to advance health for vulnerable populations. Progress in transparency and data use is evident, but accountability gaps, power imbalances, and limited engagement with civil society and private sector hinder national ownership. Further research is needed to determine GFF’s attribution to catalytic resource mobilization.

## Paper context

- **Main findings:** This multi-layered policy analysis of the Global Financing Facility for women’s, children’s, and adolescents’ health reveals US$ 14.5 billion in new funds (2015–2022) in 28 countries, mostly through World Bank lending and domestic resources, with country variability in the priorities mentioned, measured and funded and the political processes that shaped them.
- **Added knowledge:** Each layer of analysis shows unique aspects of the Global Financing Facility, starting with an investment mapping, then exploring the content of policy documents across themes, to finally understanding the policy process in four countries, showing that while it was technocratically led, it was also political, influenced by many actors, and contextual drivers.
- **Global health impact for policy and action:** Global Health Initiatives need to be held accountable for their commitments and ensure that a country-led approach is accompanied by transparent processes at every step in the policy process with broad stakeholder engagement and agreed national priorities.

## Background

Many low-income countries rely on foreign aid as part of their health-sector budget. From 2000 to 2020, development assistance for health increased 500%, with the bulk of recipients in Africa [1]. Amongst African Union countries in 2020, an estimated 10% of total health expenditure was from external financing and this varied between regions [2]. Another source put external funding at an average of 24% of total health expenditure in the whole of the World Health Organisation’s African Regional Office (WHO AFRO) region [3].

Global health initiatives (GHIs) play an important role in channelling funds for development [4]. GHIs are typically international partnerships and organisations framed around collaboratively achieving specific health goals in low- and middle-income countries (LMICs), through mobilising and disbursing funds [5]. Over 100 GHIs have been set up in the past two decades, including Gavi (the Vaccine Alliance), Global Fund to Fight AIDS, Tuberculosis, and Malaria (GFATM), Coalition for Epidemic Preparedness Innovations (CEPI), among others [6]. There are various purported benefits from GHIs, albeit with reportedly mixed successes [4], with calls for more evaluations [7]. Challenges faced by GHIs and their operational models include difficulty in tracking the flow of resources, parallel processes resulting in duplicated efforts, limited local capacity building and lack of accountability [4,6]. Further design flaws, such as lacking contextual significance, uncertain sustainability, and power imbalances, have resulted in a call for their overhaul [1].

Until 2015, there was no specific GHI aimed at increasing investment in women’s, children’s and adolescents’ health, despite the significant corresponding burden of disease. Annually, an estimated almost nine million maternal, stillbirths, children, and adolescents’ deaths occur [8,9]. Neonatal conditions rank first in the global causes of disability-adjusted life years (and fifth in the top 10 global causes of death (all ages)) [10]. Investing in women’s, children’s, and adolescents’ health not only addresses immediate health needs but also yields long-term economic and social benefits, particularly in LMICs [11]. Inadequate financing for reproductive, maternal, newborn, child, and adolescent health and nutrition (RMNCAH-N) in the highest burden countries has been a major contributor to the slow progress in mortality and morbidity reductions [12]. An analysis of overseas development aid for RMNCAH-N found an estimated US$ 15.9 billion in 2019. Despite this, neonatal deaths have consistently received the lowest investment, with < 1% of this total mentioning a specific neonatal intervention and only 0.003% mentioning any term related to stillbirths [13].

The Global Financing Facility for Women, Children, and Adolescents (GFF) was established in 2015 as the first GHI directed towards RMNCAH-N (Table 1) [14]. Driven by the recognition that traditional aid and government funding were insufficient to meet the health needs of women, children, and adolescents, the GFF aimed to support countries to develop country-led investment cases, to improve coordination of partners working on RMNCAH-N, to reform health systems and financing mechanisms, and to mobilize and align domestic resources, external financing, and private investments [15].

**Table 1.** What is the Global Financing Facility?

**Self-description** The Global Financing Facility (GFF) describes itself as ‘a country-led partnership, hosted at the World Bank, that fights poverty and inequity by advancing the health and rights of women, children and adolescents. It does this by supporting countries to strengthen health systems and improve access to care through prioritised plans, aligned public and private financing, and policy reform’ [28]. **Countries**: 36 priority countries with highest burden of deaths along the RMNCAH-N continuum **Purpose and value**: The World Bank and UN partners launched the GFF in 2015 as the ‘financing arm’ of Every Women Every Child to take forward the Global Strategy for Women’s, Children’s and Adolescents’ Health (2016–2030) [15]. It was envisioned as a way to close the US$33.3 billion funding gap, needed to meet the 2030 Sustainable Development Goals for RMNCAH-N. The GFF has multiple overlapping roles (see main functions below) and includes the GFF multi-donor trust, which is managed and monitored by the GFF secretariat hosted by the World Bank. ‘*As of June 30, 2023, the GFF Trust Fund committed a total of US$1.34 billion for GFF country grants and Essential Health Services grants in 36 countries. Of the total GFF grants committed, US$1.12 billion combined with an additional US$7.35 billion in World Bank financing, has been approved by the World Bank’s Board of Executive Directors [29]*. ’ **GFF policy documents:** There are two main documents that define the scope of the GFF engagement and that were available on their website: the investment case (IC) and the GFF-linked health financing work program, which is presented as Project Appraisal Document of the World Bank (PAD). Each is briefly described below. The GFF supports countries in the development process of the ICs and then links the GFF Multi-Donor Trust Fund grant to the World Bank funded projects [30,31]. While these documents are separate and have distinct functions, they are both country-level planning documents that are linked to the GFF.• ***The Investment Case (IC)*** describes a national strategy for women’s, children’s and adolescents’ health and defines interventions which will be funded. The investment case offers an aspirational vision but is not an operational document. The GFF has indicated that these are ‘living documents’ [30]. • ***Project Appraisal Documents of the World Bank (PAD)*** are developed by the World Bank and explain how resources will be spent over the project’s lifespan, usually five years [31]. They are country-specific and detail activities and components that will be included in a World Bank project, financed primarily by the International Bank for Reconstruction and Development (IBRD) and International Development Association (IDA), or be reflected in other donor grant operations or public budget processes. The PADs can be restructured and updated over time. Additionally, the World Bank produces annual reports on the projects. **GFF’s self-described main functions** [30]: **1. Resource mobilisation and alignment**: The GFF was designed to mobilise funding in several ways. First, countries can access GFF grants (which don’t need to be repaid) by co-financing them with loans from IDA/IBRD, meaning a country must take out a loan for an RMNCAH-N project with GFF grant money added as an incentive. Second, GFF is meant to collaborate with other in-country partners, like Gavi and the Global Fund, to align their financial resources towards shared RMNCAH-N goals, increasing efficiency and reducing duplication. Third, GFF aims to help governments increase their own spending on RMNCAH-N by providing technical assistance in managing public finances. Lastly, GFF is intended to tap into domestic and international private sector resources through mechanisms like development impact bonds. As GFF financing mechanisms are embedded within the World Bank, the GFF trust fund resources are programmed and monitored following the World Bank systems and rules. **2. Prioritising**: GFF supports countries in identifying their priority investments to achieve RMNCAH-N outcomes. In order to become a GFF partner country, the country needs to have an investment case that is evidence-based and linked to national priorities. **3. Coordinating**: GFF partners with governments to ensure the process is country-led and participatory through a country platform that enables convening with multiple stakeholders. Country platforms provide strategic direction, partner coordination, inter-sectoral collaboration, advocacy and capacity building, resources mobilisation, and monitoring and evaluation. Country platforms should include national government ministries (ideally the Ministries of Health and Finance), World Bank country staff, development partners, NGOs, the private sector, professional associations, and academic institutions. GFF also coordinates with other donors as noted under resource mobilisation. **4. Learning**: GFF emphasises the importance of learning through health system strengthening to track progress, learn and course-correct. They promote data for decision-making and have a set of core outcome and financial indicators that they track. There is focus on implementation research and delivery science within their country guidance.

Even as the GFF has become more established in the past decade, there have been few independently published analyses examining GFF commitments, investments, and processes [4,16–20]. Both independent and internal evaluations have been crucial in shaping and reforming other GHIs [4]. To address this gap, the Countdown to 2030 health Policy and Systems group formed a multidisciplinary group of academics, activists, and implementers, primarily from across Africa, to further explore the GFF policy processes [21–27]. This overview study in the Special Series [21] seeks to evaluate the GFF based on its self-described functions of resource mobilization and alignment, prioritisation, coordination, and learning (Table 1). It assesses the content and processes involved in developing two GFF country policy documents: the GFF Investment Cases (IC) and the GFF-linked World Bank Project Appraisal Documents (PADs). As part of its functions, the GFF supports countries in developing ICs. It also provides catalytic funding through its Multi-Donor Trust Fund grants which are linked to World Bank-funded projects via the PADs. The IC and PAD serve as distinct yet interconnected country-level planning tools for RMNCAH-N, but do not monitor what is actually implemented.

## Methods

This study presents multiple layers of policy analyses examining the GFF, drawing insights from the other studies in the series [21]. The first layer maps GFF country policy documents and investments. The second layer examines four tracer content analyses in these GFF country policy documents (adolescent health, maternal and newborn health (MNH), community health, and MNH care quality). The third layer compares GFF processes in four countries using an adapted health policy triangle framework. Although the three layers of analyses were conducted separately they build on each other, adding a deeper understanding of GFF policy processes.

To conduct this work, the ‘Countdown GFF policy analysis collaboration’ was established in 2021 comprising a multidisciplinary group of academics and partners, primarily from Africa. This group grew organically with intentional efforts to facilitate South–South knowledge exchange and shared learning, enable equitable partnerships, and support young and emerging academics. The group co-designed the research questions, data collection approaches, and analysis frameworks. The group held two face-to-face one-week workshops (October 2022 and March 2023) and met weekly in between to co-develop the tools, reflect on data collection, and analyse emerging themes.

### Country selection

We considered countries that had either the IC or PAD available on the GFF webpage by July 2021 or were shared by the GFF secretariat by June 2023. The four tracer content analyses (adolescent health, maternal and newborn health, quality and community) applied additional country selection criteria, detailed in those papers [24–27]. The four country case studies were selected based on the availability of both documents and the identification of in-country collaborators across different regions of Africa [21–23].

### Mapping of GFF documents

We identified documents from the GFF webpage or GFF secretariat to analyse the nature of investment. For the ICs, documents were categorized as national plans, specific investment cases with mention to GFF, or other. For the ICs, we extracted total budgets. For PADs, we extracted financial data from the ‘PAD data sheet,’ detailing the total project cost, funding sources and any financing gaps. These values were organized by PAD and country, allowing us to calculate total PAD values from the International Development Association (IDA), GFF, and other donors, along with GFF’s percentage contribution. We also reviewed PAD project descriptions, linking funding allocation by sources (IDA, GFF, or others) and summarizing the GFF-specific components, including value and project content. For PADs that did not list project components, such as those describing project restructuring, we searched for GFF mentions to determine funding allocations. These GFF-related descriptions were read and analysed for thematic similarities to identify investment focus areas. Using an inductive approach, we identified GFF’s priority investment areas. We also searched for each component of the continuum – reproductive, maternal, newborn, child, adolescent health and nutrition – to see if the terms were included in the PAD, and how these issues were framed.

### Comparison of policy content analyses of GFF documents

For the policy content analysis studies, we used the M^3^ framework – mindset, measure, money – to compare the content analyses on four thematic tracer topics: adolescent health, maternal and newborn health, community health and quality of MNH care. The framework (Additional file 1) builds from a previous exploratory content analysis of GFF country documents wherein George and colleagues examined the extent to which adolescent health was acknowledged in the programming content (mindset), indicators (measures) and investment (money) for each country’s documents. The papers in this Collection adapted this framework to each tracer theme [24–27]. To compare across the tracer themes, two authors (DK, MVK) first reviewed each study and made preliminary notes. They then summarized the findings according to the M^3^ framework and had weekly virtual meetings to facilitate discussions of cross-study insights. Finally, they identified overarching themes across each M^3^ component, documenting implications for GFF and related policy processes.

### Comparison of policy analyses in four countries

Four country case studies examining the GFF policy process (Burkina Faso, Mozambique, Tanzania and Uganda) applied an adapted Health Policy Analysis Triangle, co-developed by the collaboration (Additional file 2) [21–23]. This framework has five components – context, actors, process, content, and linkages – and was adapted to assess the policy process and power dynamics related to the GFF within countries and across them. For each component, a set of aims and questions were identified along with specific outputs expected. Data collection and analysis tools, such as the key informant interview guide and the analysis coding framework, were applied in the country studies [22,23]. The same framework was used to compare results across the four country studies. Two authors (DK, MVK) read each study report multiple times and then summarized each country’s content for the four components. Through an iterative process with the entire GFF Country Policy Analysis Collaboration, the results were further analysed and reflected on across countries, generating themes across the context, actors and process and eventually collated cross-country.

## Results

**Figure 1.**
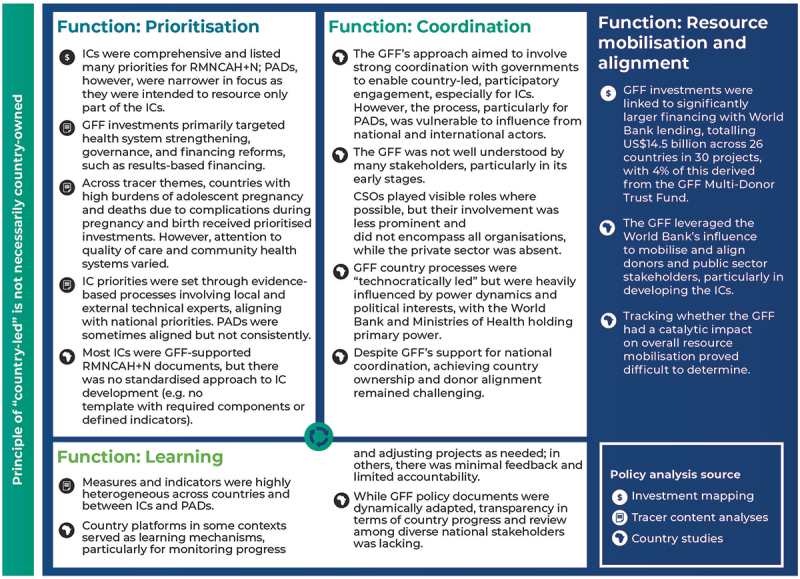
Key findings from multi-layer policy analyses mapped to the GFF framework and function areas from the GFF Country Implementation Guidelines (2019) [30]

The results are presented by the three main policy analyses undertaken: mapping GFF policy documents and investments, examining four thematic content analyses, and comparing GFF processes in four countries. Figure 1 presents an overview of our findings linked to the GFF’s main functions, described in Table 1.

### Mapping of GFF planning documents

Of the original 36 GFF priority countries, 28 countries met inclusion criteria with at least one related GFF policy document available (Additional file 4). In total, we identified 24 ICs and 30 PADs for these 28 countries (Additional file 5). Four countries did not have ICs available at the time of our study (Afghanistan, Haiti, Tajikistan, and Vietnam); and two countries did not have PADs (Madagascar and Sierra Leone). Of the 26 countries with PADs, Nigeria had three related PADs, while DRC and Liberia had two related PADs.

For the ICs, most countries had GFF-specific RMNCAH-N investment case documents that explicitly mentioned the GFF; four countries used their national health plans with no mention of GFF; two countries had summary PowerPoint presentations of their plans available; and four countries did not have published investment cases. Across the ICs, there were different priorities, indicators and budgets, often applying varying approaches and spanning different time periods, making meaningful comparison challenging.

**Figure 2.**
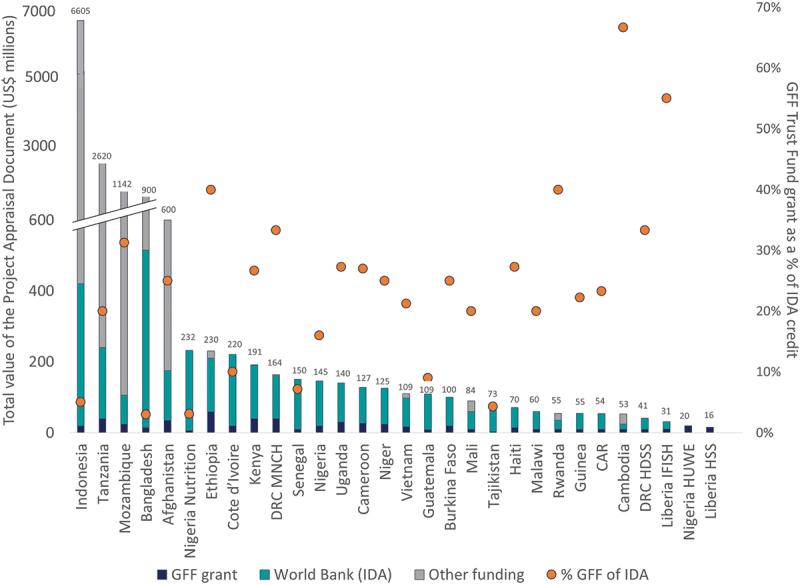
Summary of investments by country allocated through project appraisal documents.

Across the 30 PADs, the total value of funding from all sources (World Bank, GFF, domestic resources and other donors) was US$ 14.5 billion of which GFF contributed US$ 595 million − 4% of the total funds allocated (Figure 2). The highest GFF grant was US$ 60 m in Ethiopia, while the lowest was US$ 3 m in Tajikistan. Out of the total value for each PAD, the GFF contribution was lowest in Bangladesh (6% of the PAD) and highest in two PADs: Liberia Health System Strengthening and Nigeria Basic Healthcare Provision Fund Project (HUWE), where it was at 100% (as these were restructuring of existing projects). The percentage of GFF contributions compared to the overall value of the PAD (inclusive of other donors if reported) ranged between 0.3% (Indonesia) and 100% (Liberia and Nigeria restructuring documents, respectively) and had a median of 18%. When compared to the World Bank IDA value, the GFF grant ranged from 3% to 67% (Bangladesh and Cambodia, respectively) and had a median of 24%. Four PADs included the contribution from the ‘borrowing agency’ or government towards the project, which skews the total resources for these countries (Bangladesh, Indonesia, Mozambique, and Tanzania).

A thematic assessment of the PAD financial investments found common areas of focus of the GFF contribution including service delivery (i.e. providing services for RMNCAH-N, either in health facilities or in schools and communities), governance (i.e. stewardship, management, health reforms and cross-partner coordination), and Performance Based Financing. Other common areas of GFF investment included community and accountability, health systems strengthening, health workforce, and Civil Registration and Vital Statistics. Across the RMNCAH-N continuum, we found that PADs focused on different aspects, as would be expected given the different contexts and priorities. Reproductive health was explicitly mentioned but sometimes implied when there was a focus on maternal health. Maternal and child health were included in all of the PADs; however, newborns were not always explicitly mentioned [25]. Similarly, in some cases, adolescents were explicitly mentioned as being a priority group, but in others they were included only under the maternal category [26]. Nutrition was mostly addressed with regard to pregnant women and children under 5 years of age. Early childhood development was the area that had the least attention or focus across the PADS. Each PAD has a context section at the beginning that justifies the focus of the project on specific RMNCAH-N or health systems aspects. However, there is not a systematic or standard format or approach applied across the PADs that verifies that the project focus is addressing the most important priorities for the country. Detailed results of the thematic assessments are in Additional File 6.

### Comparison across tracer themes

Generally, across the four tracer themes, we observed that the ICs are comprehensive documents allowing for more priorities to be listed along with related indicators; as opposed to the PADs, which have a narrower focus on specific priorities and contain fewer indicators. In most cases, PADs aligned with a subset of components highlighted in the ICs, but there were exceptions. Applying the M^3^ framework, we found that adolescent health and MNH were given more consistent emphasis across countries and within documents, compared to community health and quality, whose content had more variation. Adolescent health was most emphasised among countries with a high adolescent pregnancy burden. However, there was mixed consistency in continuity from IC to PAD. MNH was strongly reflected and consistent in different countries, particularly antenatal care and care at birth in the ICs, while PADs focused more on Emergency Obstetric and Newborn Care and quality of care. In general, quality also faced inconsistencies between documents. While community health was acknowledged as important in all country documents, the concept varied between countries, and there was inconsistency between documents. For example, no IC in the sub-analysis undertaken had community health objectives, but they were present in PADs of Niger, Cote d’Ivoire and Guinea.

In terms of measures, the MNH and adolescent health themes had more consistent indicators. For example, most country documents applied one indicator (adolescent pregnancy) for adolescent health, and MNH had standard indicators, predominantly linked to coverage of health services, e.g. antenatal care and skilled birth attendance. Between ICs and PADs, we found fewer indicators in the PADs for MNH. Measures relating to the system themes were more inconsistent across country documents, reflecting different approaches to quality improvement and community health activities.

Relating to the money, most country ICs included a budget line for MNH, and financial allocations in the PADs were commensurate with the mortality burden for maternal, newborn and stillbirth. However, the descriptions of the funding primarily focused on interventions in pregnancy and childbirth and less so for postnatal and small and sick newborn care. Countries with a high burden of adolescent pregnancy prioritised activities related to adolescent health related interventions, with some linking adolescent-specific project components and funding. Community health and MNH-quality were included in budgets or allocations of most ICs and PADs assessed. However, not everything mentioned as a priority across these themes in the situation analysis was always reflected in the described funding. Performance Based Financing was a prominent financing mechanism overall.

### Comparison of the policy process across four countries

All the four countries included as case studies had donors supporting their health budgets in varying capacities in 2015 when the GFF started (Table 2). Across the country studies, we found variation with different contextual priorities, GFF timelines and processes, and actors engaged. However, there were some commonalities, such as policy processes were mostly led by government technocrats, GFF and World Bank teams (Additional file 6).

**Table 2.** GFF processes and investment amounts across four in-depth country case studies.

	Burkina Faso	Mozambique	Tanzania	Uganda
Timeline	2017: Process started 2018: PAD published 2019: IC published 2020: IC revised 2021: PAD restructured	2016: Process started 2017: IC drafted and circulated (April) 2017: PAD published (November)	2015: Process started 2016: IC Published (May) 2016: PAD published (May) 2021: Second IC published (November) 2022: Second PAD published (November)	2015: Process started 2016: IC published (April) 2016: PAD published (July) 2022: Second IC published
Investment amount	IC: 1,818 million US$ PAD: 100 million US$ (20 million GFF)	IC: 1,827 million US$ PAD: 105 million US$ (25 m GFF)	I^st^ IC: 1,331 million US$ PAD: 200 million US$ (40 million GFF) 2^nd^ IC: 2,078 million US$ PAD (Essential Health Services): 250 million US$ (25 million GFF)	IC: 1,918 million US$ PAD: 140 million US$ (30 million GFF)

The GFF used an adaptable, country-specific approach, tailoring policy processes to local contexts, largely prioritising RMNCAH-N through the IC process, even if this wasn’t always matched with the PADs. Countries took different approaches in the type of plans used as the IC: Tanzania used their national health sector plan as their IC in both GFF rounds; Mozambique’s IC aligned with its national health sector plan; Uganda’s IC replaced a previous RMNCAH-N plan; and Burkina Faso’s IC unified multiple plans. Existing policies and timing of the national policy cycle may have influenced this decision. For example, Tanzania had just finished their National RMNCAH-N plan (2016–2020) when GFF started working there making sense to use the same policy document as the country’s IC; whereas, Uganda developed an IC based on a revised, updated version of their Sharpened RMNCAH plan from 2013. In Burkina Faso, there had been a fragmentation of RMNCAH-N related policies and the IC process helped to align priorities and develop consensus. In Mozambique, the IC development process also enable stakeholder engagement and alignment with their national health plan. While the ICs may have helped create consensus and a unified plan, stakeholders expressed varying perspectives the utility of the ICs. With respect to the PADs, Burkina Faso and Uganda projects were solely funded by IDA/IBDR and GFF, while the PADs for Mozambique and Tanzania reported various contributions, including government, other donors, IDA and GFF.

Prioritization in the four countries was based on evidence and partner input, with GFF offering guiding principles rather than specific methods. External consultants, hired by World Bank or other partners, supported evidence generation, varying by country. GFF policy documents aligned with national policies, but also emphasized systems approaches and financial reforms, like results-based financing. Mid-term evaluations, such as in Tanzania, aided priority setting and monitoring. When included, our assessment show that Civil Society Organizations (CSOs) advocated for thematic investments in RMNCAH-N that were often overlooked by local political elites and World Bank personnel.

RMNCAH-N partner coordination varied by country with mostly Ministries of Health leading IC development and engaging multiple stakeholders. However, the processes to develop the PADs were less public and mainly led by GFF/World Bank, except in Uganda, where CSOs participated through parliamentary review mechanisms. Policy processes also evolved over time, adjusting to practical needs as seen in Burkina Faso’s PAD restructuring. Across the country studies, individual knowledge and understanding of the GFF policy processes varied among those interviewed. For some, the GFF policy processes were poorly understood, including its initiation and purpose, while others who were closer to the inner workings of the GFF processes understood the role of this GHI and that the funding allocation would be through the GFF Multi-Donor Trust Fund. There was a lack of consensus among those interviewed about the GFF’s functioning and purpose in some countries.

Power, both visible and invisible, influenced the GFF processes. In all countries, the government, mainly the Ministry of Health, led the process together with the World Bank initially. There were also other influences, including international political agendas and donors within and outside the country. The process was streamlined, timely, and better understood where there was a clear central point of seasoned, technical government leadership, exemplified by Uganda and Tanzania. High turnover of actors was a challenge in Burkina Faso, which faced multiple leadership changes due to political instability. Mozambique’s government led outwardly, but the GFF was presented as an alternative financing due to reduction of financial contributions in the previous pooled funding arrangements given the lack of donor trust.

Although GFF processes engaged diverse actors, some groups, including certain CSOs, youth, and private sector, were excluded particularly in PAD development. This sometimes led to misunderstandings and resentment by those who did not feel that they were adequately included. In the case of Burkina Faso, the IC process took longer due to deliberate efforts to engage various groups. Tanzania improved CSO involvement in the second round, benefiting from prior experiences.

## Discussion

With urgency to accelerate progress towards the Sustainable Development Goals, there are increasing calls for more targeted investment towards the health of women, children and adolescents [32]. This study examines the GFF as the first global health initiative aimed at increasing investments in these populations, unravelling the complexities of its inception and evolution through a synthesis of data from 28 countries. It includes an in-depth focus on four tracer thematic-specific analyses and four countries, employing three layers of policy analysis.

Our analyses show an incredible opportunity to advance RMNCAH-N through GFF Multi-donor trust fund commitments amounting to US$595 million in 26 countries, matched by other funds linked to the World Bank projects valued at a total of US$14.5 billion. Using a novel M^3^ framework – mindset, measures and money – we were able to look deeper into these investments by identifying if themes were prioritised, consistently addressed and resourced. We found that ICs were broader and unique to each country, while PADs had a narrower focus with fewer indicators. Adolescent health and MNH received more consistent emphasis across countries and within documents, compared to community health and quality. Alignment between ICs and PADs varied. Our third layer of analysis allowed us to go deeper since the content analysis of GFF policy documents only gave a partial view of GFF’s ability to address RMNCAH-N priorities. Findings from four country case studies on GFF policy, processes, and power found significant variability in the policy process. Though primarily technocratic, the process was also influenced by contextual factors and power dynamics.

As a vehicle for external financing, we reflect on our results considering the GFF’s main self-described functions outlined in Table 1: resource mobilisation and alignment, investment prioritisation, coordination and learning.

### Accountability in financing for RMNCAH-N

Despite a relatively small amount of funding, GFF was set up as a ‘catalytic mechanism’ to drive investment [15]. Currently, there is no independent monitoring to measure if resource mobilization (domestic or external) has improved due to GFF. Greater transparency is needed to ensure accountability to governments and constituencies [16]. Attributing improvements in health outcomes to GFF’s investments is thus difficult, causing tension as investors expect returns. Balancing these expectations underscores the debate on impact of external financing on health outcomes and alignment to national priorities [33]. Overall, more research is needed to determine the actual and perceived quantitative impact of GFF on increasing and aligning resources or possibly even displacing funding by governments or others. Our mapping of the PADs revealed variation with some projects including multiple donors, in addition to the GFF Multi-Donor Trust Fund and the World Bank, and in some cases also the contribution of the national government, but could not measure the full scope of GFF influence. Our country studies provided qualitative learnings on the dynamic and fluid nature of resource mobilization, with some donors shifting positions during negotiations.

### Prioritising

On priority setting, the four country studies found that IC priorities were determined by evidence-based processes guided by local and external technical experts, with alignment to national ‘priorities,’ as articulated in policy documents. Setting priorities has long been recognized as a complex process [34–37], especially in LMICs where weak routine data systems fail to provide accurate data for prioritisation decisions [38–40]. Additionally, evidence use for priority setting depends on the approach [41], which varied across the GFF policy documents and the country case studies. Using an existing policy for the IC versus developing a new IC could not be determined with just four country studies. While the IC development processes may have helped create consensus and a unified plan, the utility of these plans in the long-term requires more investigation. Greater clarity in GFF policy documents, particularly the PADs, on how and why specific priorities for RMNCAH-N were selected for projects would enhance transparency in the decision-making process and help other stakeholders understand opportunities and gaps.

Of course, a major challenge in the policy process is narrowing down a broad list of priorities to a focused set with consensus from stakeholders and sufficient resources for implementation. GFF investments emphasized system strengthening, including horizontal health system reforms like results-based financing, reflecting World Bank priorities [42]. It was not clear from our analyses if this priority was specific to RMNCAH-N or rather a general World Bank priority, and more research will be needed to determine if this approach is the best fit model.

Our content analyses showed that the ICs had more priorities than the PADs, which makes sense since the projects aim to support part of the IC. However, some PADs did not always align with ICs, which was unexpected since one would expect GFF-linked funds to support priorities outlined in the ICs. The inconsistency between the PAD priorities not aligning with the IC for some countries warrants further investigation and understanding. Additionally, our country studies showed that the determination of PAD priorities was less transparent and often different actors were involved in the two processes. While the PAD is a World Bank bilateral agreement with governments and has a different development process than the ICs [30,31], the different processes may have led to some of the confusion among country stakeholders, including other donors and CSOs, since the PADs do include GFF-linked resources.

### Coordinating

GFF’s approach reflected strong coordination with governments to ensure country-led engagement. CSOs played an important role where able and invited; however, they were less visible and the private sector was absent. Engaging CSO and private sector groups, which are not homogenous, is important but can be complex, costly, and time-consuming [43–45]. While little has been studied about private sector engagement with the GFF processes, the limited inclusion of CSOs in GFF processes, notably in Africa, has been documented, alongside challenges with engaging CSOs meaningfully and country platform functionality [19]. There have been intentional efforts by GFF to coordinate better with civil society, including through the establishment of the Global Civil Society Coordinating Group for the GFF and a CSO Hub [46,47], though the impact of these new structures has yet to be systematically assessed across countries.

This multi-layered study showed that the World Bank, GFF, and Ministries of Health held the most power through primarily technocratically-led processes. The technical nature of documents in some instances made the GFF policy processes inaccessible to civil society and development partners alike. In other studies, domination of medical professionals in the health policy process results in more focus on biomedical or clinical approaches than systems approaches that require multidisciplinary solutions [48]. Political dynamics also influence these processes with donor power shaping priorities and outcomes. Donor influence has been noted elsewhere in policy formulation, as has power imbalance between actors [49,50]. The perceived and observed link between GFF and the World Bank likely had its advantages and disadvantages for the GFF’s ability to convene; but importantly, it demonstrates the power imbalance that donors, such as World Bank, have in influencing national priorities [49].

There was variable success in ensuring government and national ownership in terms of process. Ownership has not been clearly defined [51], and it is hard to distinguish between ‘owned’ and ‘led’, especially for RMNCAH-N, where the needs of marginalised populations need to be met. The World Bank assesses ownership by evaluating six components, including civil society and private sector engagement [52]. However, a checklist alone may not capture the complexity of ownership. Clear guidelines that address the politics of change and social justice within RMNCAH-N are needed, as governments, health professionals, and donors may or may not actively advocate for marginalised mothers and newborns.

### Learning

For the GFF’s role in learning, country platforms play a critical role in this space, enabling course correction as needed and reflecting a policy learning system [53]. While the GFF has a set of core impact indicators and finance indicators [30,54], these were not routinely reflected in the country documents. Indicator shortlists can help tracking over time and between countries, and support multi-country learning [55]. Global health metrics is a political space [56] and there is a challenge of multiple competing RMNCAH-N measurement frameworks. GFF has a role in harmonizing and translating them for country application. Our results also reflected known global RMNCAH-N measurement gaps, such as experience of care [57]. There is also a need to invest in national routine information systems and data use, not just Civil Registration and Vital Statistics. High impact indicators, such as stillbirth rates, remain absent in many national policies despite calls for inclusion [58]. GFF plays a role in developing priority indicators for consistent tracking across countries [55]. The M^3^ framework may be a useful tool to monitor whether stated priorities are measured and funded within the GFF process [24–27].

GFF has demonstrated how a GHI can be responsive and adaptive, as noted previously on the CSO engagement activities. Other broader reforms, such as addressing gender norms, were also an acknowledged gap during the first phase and the new GFF strategy puts these issues as core elements of their new strategy [59]. The GFF has made concerted efforts to support countries through the GFF Knowledge and Learning Platform, which is a rich resource database and engagement platform [60]. The GFF also promotes annual reviews, including within countries and as a global initiative [54], and enables project restructuring in response, as seen in Burkina Faso. Our analysis of GFF policy processes covered its initial phase, when these elements had not been put in place. Further assessments are needed to review the effectiveness and perceptions around the GFF technical assistance provided to support the learning agenda.

### Study strengths and limitations

This study has several strengths, among which is the multi-country, multi-method approach. We included 28 of the 36 GFF countries, based on document availability, and four country studies, resulting in a large dataset. While this was a convenience sample and limited by the information available at the time, it included significant regional diversity and drew from significant country-based expertise in RMNCAH and health systems.

The variety of approaches brought a deeper analysis of the individual topics assessed. The study applied frameworks supported by protocols and tools, enabling comparability between studies. For this paper, rigour was ensured through double data extraction and repeated review of study papers.

The study was conducted by a collaborative, diverse, multi-disciplinary team mainly in and from the countries studied, supported by an equitable partnership agreement. Regular collaborative meetings and data analysis workshops were held. Importantly, there was reflective learning across topics and countries, which strengthened analysis and discussions on results’ implications. Stakeholder engagement with the GFF Secretariat and in-country actors helped clarify documents and validate findings.

Given our assessment was of a new mechanism that was still growing, fundraising and adapting its approaches, some GFF targets shifted, as did availability of GFF documents during the study period. The study primarily focused on the first GFF funding cycle and thus looked at initial documents and policy processes, mostly from before 2020. Funding and time capacity constraints limited the breadth of our work overall; for example, we could only conduct four country case studies. We also primarily assessed the GFF policy documents (ICs and PADs), which are aspirations or planning documents and do not track implementation. We did not assess the impact of the GFF in mobilising and aligning resources, which, while important, was beyond the scope of this study. Table 3 lists some future research questions that might be useful for further understanding the role and potential of GFF in advancing RMNCAH-N.

**Table 3.** Illustrative future research questions.

Did the GFF catalyse resource mobilisation? How can the increases in resource mobilization be better measured, also with consideration of displacement of government funding? What is the GFF’s role in setting research priorities especially for investment, and sustainable financing leading to impact? How does a country move from a long list of priorities in an Investment Case to ‘priorities of priorities’, while ensuring meaningful inputs from diverse stakeholders? What is ‘country ownership’ in practice and how can this be measured and promoted for RMNCAH-N? How can the diversity of actors be engaged for better ownership? What can be learnt from other funding bodies? What is the GFF’s role in democratising implementation research frameworks/methods and enable multi-county learning networks? How can the implementation of IC and PADs be measured, to ensure that money is accessed by the right actors and spent, is aligned to priorities, and actually makes a difference? How have the technical assistance processes supported by the GFF enabled further country deliberations and contribute to aligning technical assistance? Are these at the expense of more transparent and inclusive processes? What does it entail for broader accountability of donor assistance and government leadership? Can modelling, e.g. Lives Saved Tool, assess plausible impact and contribution of a GHI to health outcomes? How can GFF be held accountable to citizens, not just Ministries of Health?

## Conclusion

The GFF is linked to much greater financing with World Bank lending, but to date there is no independent monitoring to assess overall effects on resource mobilization levels or trends. By leveraging World Bank convening power, the GFF has an important role in supporting countries to set priorities, coordinate and advance the learning agenda. The four in-depth country case studies found that while IC priorities were determined with input from local and external experts, there were challenges in moving from broad lists and national sector plans to actionable, resourced priorities. GFF’s coordination approach ensured country-led engagement with governments. Although it was technically led, it had initially limited inclusion of civil society and private sector actors. Power imbalances between the World Bank, Ministries of Health and donors complicated processes and shaped outcomes, highlighting challenges in achieving true national ownership, donor coordination, resource mobilisation and stakeholder engagement. Future research is needed to measure the catalytic impact of GFF in advancing women’s adolescents and children’s health to support its transformative aims.

## Supplementary Material

Supplemental Material

## Data Availability

The datasets used and/or analysed in this study are available in the additional files or from the corresponding author on reasonable request.
