# Supplementary material for: Unwrapping the Global Financing Facility: understanding implications for women’s children’s and adolescent’s health through layered policy analysis
Source: Glob Health Action. 2025 May 16;18(1):2476820. doi: 10.1080/16549716.2025.2476820 (PMC12086900; doi:10.1080/16549716.2025.2476820)
Supplement: Supplemental Material [file ZGHA_A_2476820_SM6330.docx]

**Title**: Unwrapping the Global Financing Facility: Understanding implications for women's children's and adolescent's health through layered policy analysis

Contents

[Additional File 1: M3 Framework used for policy content analysis approach 1](#_Toc182237271)

[Additional file 2: Framework for the policy process, people and power case study approach 2](#_Toc182237272)

[Additional file 3: Country selection 2](#_Toc182237273)

[Additional file 4: Mapping GFF country documents 3](#_Toc182237274)

[Additional File 5: Results of mapping investments (US$) 5](#_Toc182237275)

[Additional file 6: Analysis tables 8](#_Toc182237276)

**Additional files**

## Additional File 1: M3 Framework used for policy content analysis approach


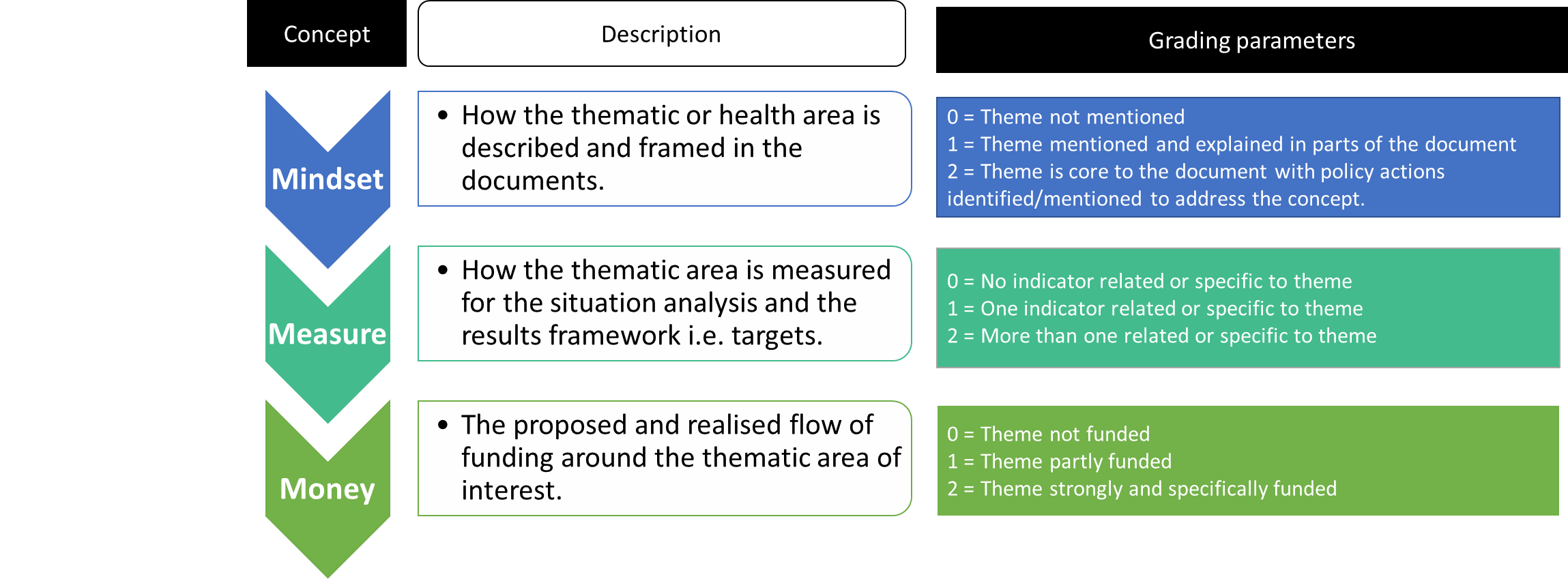


## Additional file 2: Framework for the policy process, people and power case study approach


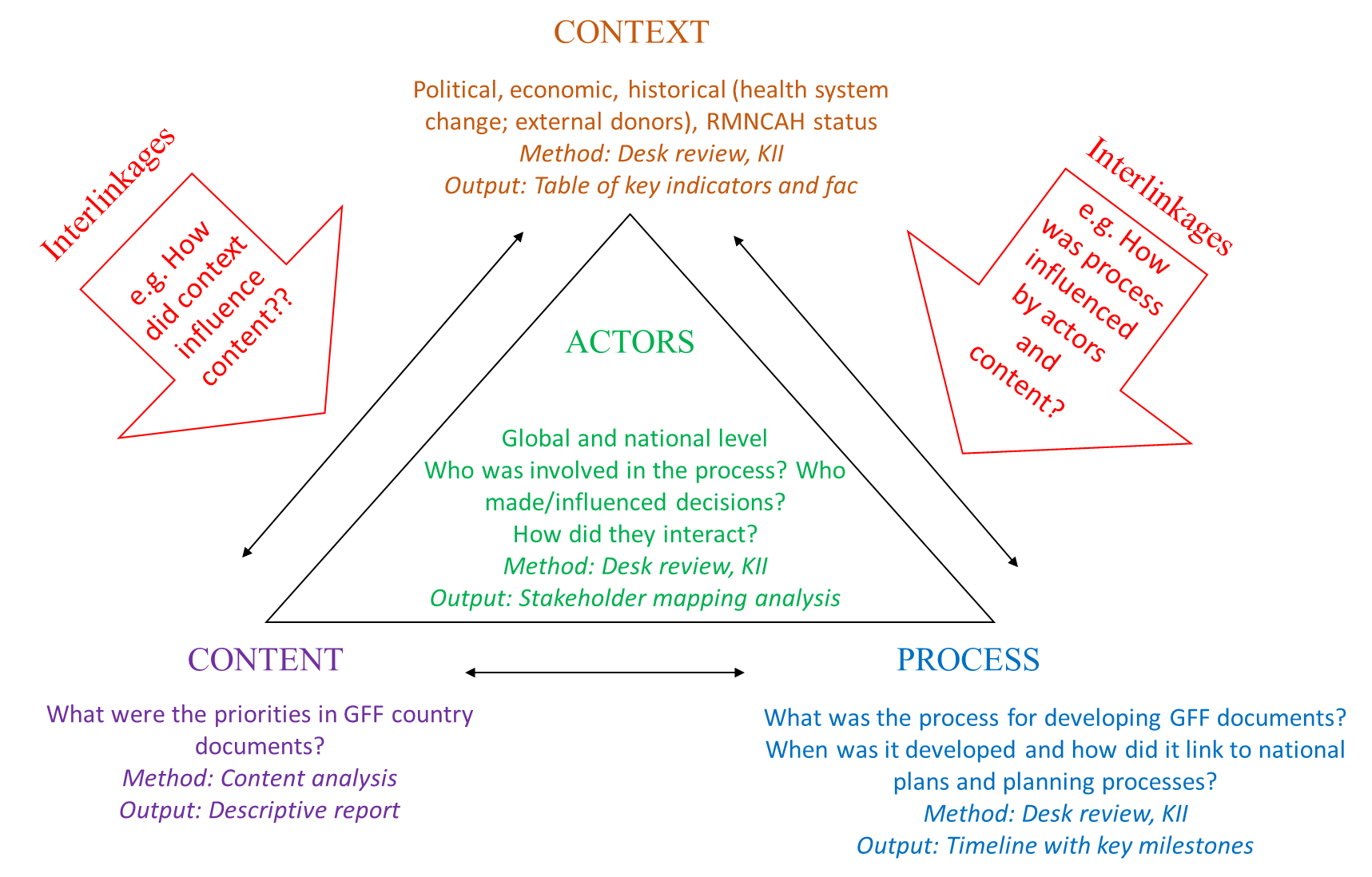


## Additional file 3: Country selection


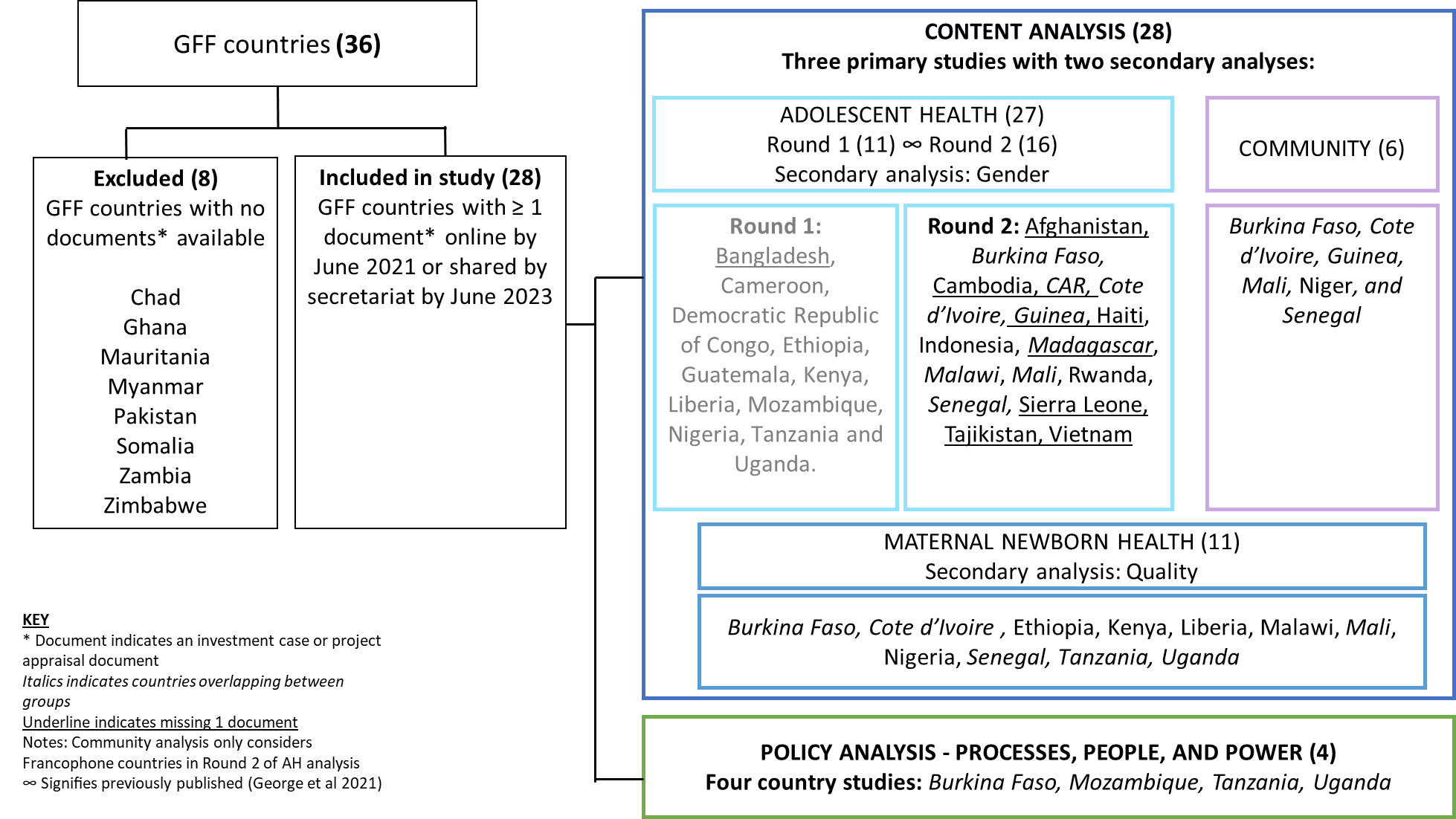


## Additional file 4: Mapping GFF country documents

|  | **Country** | **IC** | **TYPE OF IC** | **SOURCE** | **IC Language** | **PAD*** | **SOURCE** | **Number of documents** | **NO DOCS** | **# IC** | **# PAD** |
| --- | --- | --- | --- | --- | --- | --- | --- | --- | --- | --- | --- |
|  |  |  |  |  |  |  |  |  |  |  |  |
| 1 | Afghanistan | NO | None | NO DOCUMENT CONFIRMED BY SECRETARIAT | English | YES | WEBPAGE | 1 | 0 | 0 | 1 |
| 2 | Bangladesh | YES | National plan | WEBPAGE | English | YES | WEBPAGE | 2 | 0 | 1 | 1 |
| 3 | Burkina Faso | YES | Specific IC | WEBPAGE | French | YES | WEBPAGE | 2 | 0 | 1 | 1 |
| 4 | Cambodia | YES | PPT summary specific IC | SECRETARIAT | English | YES | WEBPAGE | 2 | 0 | 1 | 1 |
| 5 | Cameroon | YES | Specific IC | WEBPAGE | French | YES | WEBPAGE | 2 | 0 | 1 | 1 |
| 6 | CAR | YES | Specific IC | WEBPAGE | French | YES | SECRETARIAT | 2 | 0 | 1 | 1 |
| 7 | Chad | NO |  | NO | N/A | NO | N/A | 0 | 1 |  |  |
| 8 | Cote d’Ivoire | YES | Specific IC | WEBPAGE | French | YES | WEBPAGE | 2 | 0 | 1 | 1 |
| 9 | DRC | YES | Specific IC | WEBPAGE | French | YES | WEBPAGE | 3 | 0 | 1 | 2 |
| 10 | Ethiopia | YES | National plan | WEBPAGE | English | YES | WEBPAGE | 2 | 0 | 1 | 1 |
| 11 | Ghana | NO |  | NO | N/A | NO | N/A | 0 | 1 |  |  |
| 12 | Guatemala | YES | PPT summary specific IC | WEBPAGE | English | YES | WEBPAGE | 2 | 0 | 1 | 1 |
| 13 | Guinea | YES | Specific IC | WEBPAGE | French | YES | SECRETARIAT | 2 | 0 | 1 | 1 |
| 14 | Haiti | NO | None | NO DOCUMENT CONFIRMED BY SECRETARIAT | N/A | YES | WEBPAGE | 1 | 0 | 0 | 1 |
| 15 | Indonesia | YES | National plan | SECRETARIAT | English | YES | SECRETARIAT | 2 | 0 | 1 | 1 |
| 16 | Kenya | YES | Specific IC | WEBPAGE | English | YES | WEBPAGE | 2 | 0 | 1 | 1 |
| 17 | Liberia | YES | Specific IC | WEBPAGE | English | YES | WEBPAGE | 2 | 0 | 1 | 2 |
| 18 | Madagascar | YES | Specific IC | SECRETARIAT | French | NO | N/A | 1 | 0 | 1 | 0 |
| 19 | Malawi | YES | Specific IC | SECRETARIAT | English | YES | SECRETARIAT | 2 | 0 | 1 | 1 |
| 20 | Mali | YES | Specific IC | WEBPAGE | French | YES | WEBPAGE | 2 | 0 | 1 | 1 |
| 21 | Mauritania | NO |  | NO | N/A | NO | N/A | 0 | 1 |  |  |
| 22 | Mozambique | YES | Specific IC | WEBPAGE | English | YES | WEBPAGE | 2 | 0 | 1 | 1 |
| 23 | Myanmar | NO |  | NO | N/A | NO | N/A | 0 | 1 |  |  |
| 24 | Niger | NO | Specific IC | Yes | French | Yes | WEBPAGE | 2 | 0 | 1 | 1 |
| 25 | Nigeria | YES | Specific IC | WEBPAGE | English | YES | WEBPAGE | 4 | 0 | 1 | 3 |
| 26 | Pakistan | NO |  | NO | N/A | NO | N/A | 0 | 1 |  |  |
| 27 | Rwanda | YES | Specific IC | WEBPAGE | English | YES | WEBPAGE | 2 | 0 | 1 | 1 |
| 28 | Senegal | YES | Specific IC | WEBPAGE | French | YES | WEBPAGE | 2 | 0 | 1 | 1 |
| 29 | Sierra Leone | YES | Specific IC | WEBPAGE | English | NO | NO RESPONSE FROM GFF FOLLOW UP | 1 | 0 | 1 | 0 |
| 30 | Somalia | NO |  | NO | N/A | NO | N/A | 0 | 1 |  |  |
| 31 | Tajikistan | NO | None | NO DOCUMENT CONFIRMED BY SECRETARIAT | N/A | YES | WEBPAGE | 1 | 0 | 0 | 1 |
| 32 | Tanzania | YES | National plan | WEBPAGE | English | YES | WEBPAGE | 2 | 0 | 1 | 1 |
| 33 | Uganda | YES | Specific IC | WEBPAGE | English | YES | WEBPAGE | 2 | 0 | 1 | 1 |
| 34 | Vietnam | NO | None | NO DOCUMENT CONFIRMED BY SECRETARIAT | N/A | YES | WEBPAGE | 1 | 0 | 0 | 1 |
| 35 | Zambia | NO |  | NO | N/A | NO | N/A | 0 | 1 |  |  |
| 36 | Zimbabwe | NO |  | NO | N/A | NO | N/A | 0 | 1 |  |  |

* all PADs available in English

Acronym list: IC – investment case; PAD – Project Appraisal Document, CAR – Central African Republic; DRC – Democratic Republic of Congo

## Additional File 5: Results of mapping investments (US$)

| Country | Year | Total PAD value (US$ millions) | Other funding (not GFF or IDA) (US$ millions) | GFF grant (US$ millions) | World Bank contribution (IDA/ IBRD) (US$ millions) | GFF as a % of IDA* | GFF as % of total PAD value | GFF focus summary |
| --- | --- | --- | --- | --- | --- | --- | --- | --- |
| Afghanistan | 2018 | 600 | 425 | 35 | 140 | 25% | 6% | Improving service delivery ($28m), health system strengthening ($5m), Demand and community accountability ($2m) |
| Bangladesh | 2017 | 900 | 385 | 15 | 500 | 3% | 2% | Service delivery specific to postpartum family planning ($4.5), infant and child nutrition ($5m), and school based adolescent health programme ($5.5m) |
| Burkina Faso | 2018 | 100 | 0 | 20 | 80 | 25% | 20% | Health system strengthening ($10m), MNCAH service delivery ($5m), CRVS ($2) |
| Cambodia | 2019 | 53 | 28 | 10 | 15 | 67% | 19% | Service delivery (ECD) ($2m), demand & accountability at community level ($2.6m), effective response & sustainability ($5.4m) |
| Cameroon | 2016 | 127 | 0 | 27 | 100 | 27% | 21,3% | Health system strengthening including PBF and improving access to service delivery ($25m); strengthening CRVS ($2m) |
| CAR | 2018 | 54 | 1 | 10 | 43 | 23% | 19% | Service delivery through PBF ($7m), health system strengthening & gender based violence ($3) |
| Cote d’Ivoire | 2019 | 220 | 0 | 20 | 200 | 10% | 9% | Strategic purchasing - PBF and National Health Insurance health reforms and capacity building ($5m), governance and strengthening HMIS & CRVS ($15) |
| DRC – Human development systems strengthening project | 2016 | 41 | 1 | 10 | 30 | 33% | 24% | CRVS ($10m) |
| DRC - Health system strengthening for better maternal health and child health results project | 2017 | 164 | 4 | 40 | 120 | 33% | 24,5% | PBF focus with aim to strengthen health services ($12m), improve governance ($5m), and strengthen health sector performance financing and policy including PBF, human resource, commodities/supply chain, guideline and healthcare provision ($23m) |
| Ethiopia | 2017 | 230 | 20 | 60 | 150 | 40% | 26% | Program for results (including MNH) ($51.5m), CRVS ($5), Nutrition ($2.5m), Unallocated ($1) |
| Guatemala | 2017 | 109 | 0 | 9 | 100 | 9% | 8,26% | Buydown on the interest and/or other loan charges upon achieving the agreed indicators ($9m) |
| Guinea | 2018 | 55 | 0 | 10 | 45 | 22% | 18% | Service delivery including commodities & access to water and electricity and district health strengthening linked to PBF ($5m), community health workers ($2m), MOH financing capacity & reform PBF ($2m), donor coordination ($1m) |
| Haiti | 2019 | 70 | 0 | 15 | 55 | 27% | 21% | Service delivery ($12m), infectious disease ($2m), project management & implementation support ($1m plus TA) |
| Indonesia | 2018 | 6605 | 6185 | 20 | 400 | 5% | 0,30% | Institutional strengthening and coordination ($16m), district capacity strengthening ($4m). |
| Kenya | 2016 | 191 | 1 | 40 | 150 | 27% | 21% | PHC including MNH interventions ($35m), Strengthening Institutional Capacity ($5m) |
| Liberia – Health System Strengthening restructure | 2017 | 16 | 0 | 16 | 0 |  | 100% | Support to Quality Service Delivery Systems ($13.7m) Support to Strengthening Fit-for-Purpose Health Workforce ( $0.5m) Project Management ($1.2m), Health system strengthening post Ebola ($0.6m) |
| Liberia -Liberia Institutional Foundations to improve services for health projects “IFISH” | 2022 | 31 | 0 | 11 | 20 | 55% |  | Service delivery specific to community and adolescent health care ($3m), human resource strengthening ($1m), PBF ($5m) and project coordination ($0.50m) |
| Madagascar |  |  | 0 |  |  |  |  | n/a (no PAD) |
| Malawi | 2018 | 60 | 0 | 10 | 50 | 20% | 17% | Not specified |
| Mali | 2019 | 89 | 29 | 10 | 50 | 20% | 11% | Service delivery ($4m), Community activities ($3m), stewardship and CRVS ($1.5m) |
| Mozambique | 2017 | 1142 | 1037 | 25 | 80 | 31% | 2,19% | not specified |
| Niger | 2021 | 125 | 0 | 25 | 100 | 25% | 20,00% | Service delivery, utilization, quality and supply ($15m); Health and nutrition demand creation and services ($10m) |
| Nigeria - Nigeria State Health Investment Project (NSHIP) | 2016 | 145 | 0 | 20 | 125 | 16% | 14% | Service delivery including MNH ($14m), Health system strengthening ($6m) |
| Nigeria “HUWE project” | 2018 | 20 | 0 | 20 | 0 |  | 100,00% | Financial and management support to financing mechanisms (eg fee-for-service) aimed at strengthening primary health care ($20m) |
| Nigeria Nutrition | 2018 | 232 | 0 | 7 | 225 | 3% | 3,02% | Nutrition ($5.6m); stewardship and project management ($1.4m) |
| Rwanda | 2018 | 55 | 20 | 10 | 25 | 40% | 18% | CHW program & district multi-sectoral response ($8m), learning, knowledge sharing and M&E ($2m) |
| Senegal | 2019 | 150 | 0 | 10 | 140 | 7% | 7% | Health workforce skills ($5m), Governance ($5m) |
| Sierra Leone |  |  | 0 |  |  |  |  | n/a (no PAD) |
| Tajikistan | 2020 | 73 | 0 | 3 | 70 | 4% | 4% | Service delivery ECD ($3m) |
| Tanzania | 2015 | 2620 | 2380 | 40 | 200 | 20% | 2% | Not specified |
| Uganda | 2016 | 140 | 0 | 30 | 110 | 27% | 21% | Results based financing for PHC ($25m), CRVS ($5m) |
| Vietnam | 2019 | 109 | 12 | 17 | 80 | 21% | 16% | Not specified |
| **TOTAL** |  | **14526** | **10528** | **595** | **3403** | **17%** | **4%** |  |

* The GFF grant aimed to be around 20% of the IDA/IBRD for supported projects according to two consultants who worked with GFF and were interviewed for two different country studies. We could not find documentation to validate this.

Acronym list:

CAR: Central African Republic

CHW: Community Health Workers

CRVS: Civil Registration and Vital Statistics

DRC: Democratic Republic of Congo

ECD: Early Childhood Development

HMIS: Health Management Information System

IBRD: International Bank for Reconstruction and Development

IC: investment case

IDA: International Development Association

M&E: Monitoring and Evaluation

MNCAH: Maternal, Newborn, Child, and Adolescent Health

MNH: Maternal and Newborn Health

MOH: Ministry of Health

PAD: Project Appraisal Document

PBF: Performance-Based Financing

PHC: Primary Health Care

TA: Technical Assistance

## Additional file 6: Analysis tables

Table 6.1: Cross country study analysis

|  | Burkina Faso | Mozambique | Tanzania | Uganda | SO WHAT |
| --- | --- | --- | --- | --- | --- |
| Timeline | 2017 - Process started  2018 -PAD published  2019 - IC published  2020 - IC revised  2021 - PAD restructured | 2016 – IC developed  2017 – PAD published | 2015- Process started  2016- IC Published (May)  2016- PAD published (May)  2021- Second IC published (Nov)  2022 – Second PAD published (Nov) | 2015 - Process started  2016 -IC published (April)  2016 - PAD published (July)  2022 – Second IC published | Different processes – but most countries had IC before PAD. |
| Investment amount | IC – 1,818 million US$  PAD – 100 million US& (20m GFF) | IC – 1827 billion US$  PAD – 105 million US$ (25m GFF) | I^st^ IC – 1331 billion US$  I^st^ PAD – 200 million US$ (40m GFF)  2^nd^ IC – 2078 billion US$  2^nd^ PAD- 250 million US$ (25m GFF) | IC - 1,918 million US$  PAD – 140 million US$ (30m GFF) | BF and UG similar IC budgets; MZ and TZ similar IC budgets  PADs ranged from 100m to 250m |
| Main results (themes) | Strong political prioritization of RMNCAH-N was undercut by fragmentation in financing and delivering on these goals   - Government committed & took actions including co-hosting event plus waiving some payments - Competing policies were a problem   Negotiating different interests and misunderstandings   - Some had more power due to technical, financial strength and seniority - Pressure to meet some deadlines e.g. PAD 1 - Confusion around GFF funding model negatively affected motivation   Document development processes were consultative, involving a range of national and international actors  Implementation of the IC and PAD was not institutionally led or supported by all key stakeholders   - Unclear leadership in MOH for IC amidst high turnover in MOG thus multiple documents and donors. - PAD also suffered poor ownership - Lack of consensus on SHP | GFF arrived in stormy context (debt scandal, donors fragmented and leaving)  Different types of power and legitimacy: GFF led by the World Bank (not government or country led)  GFF gathered donors around PHC-SP after a period of distrust and pulling out  Resources for health have increased overall since the initiation of the GFF | Political landscape at the time meant there was support for GFF at global & national levels  Government owned and led process, but DLIs mechanism, whilst improving accountability, meant WB held hidden power  Involvement of key stakeholders moved during the process, through closed and invited spaces - though served to bring people together with same mandate  Overall successes, challenges & recommendations: GFF mechanism enabled an environment where efficient planning could take place for RMNCAH services knowing funding relatively secure | GFF documents process unfolded rapidly, building on existing policy documents and processes (eg TWG)  It was largely country-led though the process was also influenced by global actors via CSOs and the “elites”  (Mis)understanding of GFF by multiple actors (including those within government) due to limited engagement in the development of the PAD  Different interests of actors were revealed especially between technical and political actors and global influence   - IC content shaped by evidence, plus technical & political actors - PAD content shaped by technocrats & politicians |  |
| Content | Alignment between documents  Focus is on strengthening health system  Combined/collated previous plans | Alignment between documents with some distinctions/ focus areas in PAD  Content built from or linked to previous policies  IC is a broad costed implementation strategy  The PAD focuses on improving the utilization and quality of RMNCAH and nutrition in underserved areas through the project called “PHC Strengthening Program” (PHCSP). | Investment Cases 1 and 2 in Tanzania are National Strategic Plans for maternal, newborn, and child health (One Plan II and One Plan III).  PAD 1 was developed by the Consultant on behalf of the government. PAD 2 was developed collaboratively between the government officials and other RMNCAH-N key stakeholders    The document review found that gender norms were mentioned with regard to SRH for women and adolescents (linked to GBV) though gave less focus on health systems  Excluded stillbirths, family planning, people with disabilities and boys’ SRH | Alignment between documents with some distinctions  Content built from or linked to previous policies  Aligned with existing national & global policies, thus some continuity in content  Areas of focus:  Holistic approach, focusing on improving service delivery and health system aspects, with a particular emphasis on human resources (specifically midwives); Results-based financing mechanisms;  Improving care quality;  CRVS; MPDSR | Policy content in GFF documents built from or linked to previous or existing policies. There was mostly alignment between the ICs and PADs in terms of priorities |
| Context | Volatile socio-political environment and terrorism  International commitments and national strong political support to maternal and child health and to the GFF  Fragmentation of RMNCAH policies and financing at national level with a long list of ‘competing’ policies, strategies, and plans related to RMNCAH-N  Various financing mechanisms for RMNCAH in the country  High turnover in the Ministries of health and finance, as well as at the World Bank  Country was dependent on foreign aid | Donor dependency  Financial scandal  Post-conflict | Alignment between GFF goal and Tanzanian government vision to meet SDGs. Political will present  Tanzania one of only four of the front runner countries  Sharpened One Plan was launched by Former President Kikwete.  IC is framed around SGDs and notes slow progress on maternal mortality  A concept note was written by Government showing priorities  MoH & WB signed MOU guided by the HSSP IV and One plan III (priorities) & WB supported the areas of interest  Tanzania uses a SWaP mechanism for pooling resources into health basket fund  High domestic financing  Political support for GFF existed nationally & created enabling environment | Despite progress in RMNCAH, Uganda still has a high burden of maternal & child mortality  GFF came in at the time of presidential & parliamentary elections. Political rulers remained the same  Health minister changed along the way but new one was already in the system so not much change  Country has a heavily donor financed health sector | Donors play a major role in these contexts – different processes in different countries e.g. Tanzania manages through a SWAP, Mozambique had lost donor trust  Political transitions through elections or shifting roles of key actors had different impacts on the process. |
| Actors | Leader: MOH & WB  (for both IC & PAD?)  High turnover of leadership in MOH  A plethora of implementing agencies and 26 national and international donors were working in the country using a variety of funding mechanisms   - Funders influenced priorities and had their own preferences - Consultants used for IC - 4 technical working groups for IC - Other ministries also involved in IC, creating new contacts for cross-sectoral work - Actors had varied power | Leader: WB  World Bank was perceived as the “gorilla in the room” and the Project Document Appraisal as “the only game in town” | Leader: MOH & WB (During the first round, the power remained heavily with the WB, GFF and MoH through decision making in closed spaces). Some closed spaces even within government  Actors able participated within decision making spaces shifted through time, and across the two waves of the GFF.  CSOs in the GFF process and opportunity to participate were more limited. Through the early stages CSOs were not involved  Lack of involvement of the private sector; RMNCAH -N multilateral partners involved  Different actors between IC and PAD  Different actors with different power. E.g. hidden power by World Bank  Technical support from consultants | Leader: MOH (for IC)  CSOs involved but not all and contested  Country-led approach by government and other actors (technocrats) and some political figure engagement: Existing RMNCAH TWG with diverse representation  Backstage role and influence of some global players, through CSOs  Had some instances of politicians versus technocrats | Multiple actors included in IC development  Often fewer actors involved in PAD development   - - Tanzania PAD 1 done by 1 consultant - Different actors involved in IC and PAD (constant actors in both were MoH and World Bank/GFF) - Certain important actors that were excluded or not meaningfully engaged   - Private sector, youths, citizens, CSOs, women’s groups, etc - Actors included either due to their roles or their power (or both) - Power of actors took various forms (Tz) |
| Process | - Consultative process for both IC and PAD - Inclusive - Evidence-based (SMART,SARA, GBD etc) - There were smaller advocacy groups at play, some supported by donors - Multi-sectoral / very inclusive approach taken for IC made it go slower than planned - Deliberately sought out other players for inclusivity - IC used pre-existing platform | IC had a very consultative process but the PAD had no external dialogue or consultation  Main platform (SWAP thematic working group) had dissolved. | Main platform: The national strategic documents serve as the IC & SWAP, TWGs, other government departments, and partners. Used already existing country strategy (One Plan II)  Costing of One Plan II was done using Lives Saved Tool (LiST) and UN One Health Costing Tool  PAD 1&2 were prioritised using the National Health Sector Strategic Plan IV and V  The national documents were developed separately, engaging key national stakeholders, including RMNCAH technocrats.  WB used consultants in the process of developing a concept note to identify interventions for funding.  Country led but power in Ministry  Evolvement over time, PAD 2 was more collaborative - PAD 1 by WB consultant  Evidence based | Main platform: the already existing RMNCAH TWG – multiple partners, led by skilled MoH team    IC developed with many partners through TWG  PAD negotiated through separate structure (MoF and parliament)   - Rapid process - Used various prioritization tools for IC   (LiST; Bottleneck Analysis)   - Smaller group - Less definition of process in PAD. Most from interviewees - Policy development was aligned with previous documents - Did not create a new structure for the IC development process but chose to use and expand an existing functional platform | - Document development was typically a government-led process - PAD typically done after IC (except Burkina for the 1^st^ set) - Engagement of different groups of people   - Though in some cases there was contradictory information e.g. for CSOs - Use of evidence to prioritize what to focus on - Some cases like Burkina affected by insecurity - Some used a multi-sectoral approach / view, for instance considering the education sector - No need to create new structures if you already have existing ones that can be modified to suit a new process |
| Interaction | High turnover among leadership of MOH prevented sustained political attention to the IC and its adoption by all stakeholders, especially those who were not part of the process from the outset  Insecurity and political instability contributed to observed delays and difficulties in implementing the commitments agreed upon  Some donors continued to fund RMNCAH outside the IC and through other routes. So IC was not always used for its intended purpose | The PAD process helped reconvene partners to join the multi-donor trust to support PHCSP  Donor and government resources for health increased after the introduction of GFF | Need a more inclusive process from the start. Just because some groups lack technical expertise or financing does not mean they do not have valuable knowledge to contribute to planning and funding use e.g community, CSOs, private sector but also members of parliament and key decision makers  Focus on meaningful engagement with the community - highlighted as a key gap | GFF documents were broadly shaped by the use of evidence and the influence of technical and political actors  Exclusion of some important groups initially led to a mistrust and confusion around the GFF funding mechanism  CSOs are a significant force in policymaking because they wielded influence in the parliamentary, cabinet and health sectors that none of the other actors did; They also have ties with global partners |  |

Table 6.2: Cross thematic content analysis

|  | AH | MNH | Quality | Community | SO WHAT |
| --- | --- | --- | --- | --- | --- |
| # of countries | 16 | 11 | 11 | 6 |  |
| # of documents | 27 | 24 | 24 | 12 |  |
| MAIN FINDING | Topic generally strongest in countries where needs are greatest. However, some fragile settings were an exception, barely focusing on AH despite the high burden  Topic mostly focused on one aspect i.e. reproductive health (teen pregnancy)  Topic in ICs was often not carried through to PADs. Some countries demonstrate how to integrate AH well in both documents (BF, Malawi).  Findings were similar to round 1, with mostly higher burden countries placing more focus on AH  AH was addressed mostly in mindset, less in measure and least in money | Topic strongly reflected in the documents  There are some gaps and opportunities to strengthen MNH policy content and better align burden with investments, notably for stillbirth and programme areas beyond care at birth, notably neonatal care.  Investment commensurate with burden of MNH mortality.  Focus though was mostly on pregnancy and birth and how to improve and finance interventions  Content lessened between ICs and PADs  GFF provides a good opportunity to include MNH policy and to have smart investments grounded in country context | Topic is of interest and broadly included in documents but not systematically focused on MNH across countries.  Health systems aspects are often mentioned and funded but disconnected from MNH and the continuum of care | Topic acknowledged as an integral part of the health system and GFF stimulating the development of community health strategic plans in countries  Financial payments or incentives were more often mentioned in PADs than in ICs, and in PADs usually through PBF  CH mentioned but it is fragmented  PADs sometimes had issues not picked from ICs, thus not harmonised  Multiple definitions, thus hard to define scope  GFF can catalyse addressing CH through deliberate focus on it in the documents |  |
| Mindset | - Adolescents are mentioned to some extent in most country documents but are inconsistently featured across the ICs and PADs. - No formal definition given though vulnerability of adolescents emphasised - Only Burkina Faso & Malawi had strong AH focus from IC to the PAD - Mali & CAR: strong in ICs not PADs - AH services described mostly SRH, GBV, HIV, FGM, abortion, adolescent friendly services - Little mention of Boys except in CAR and Guinea - Few addressed engaging adolescents in planning | Maternal and newborn health care packages strongly mentioned, especially for pregnancy and childbirth. Some significant gaps in content (stillbirths, midwives, small and sick newborn care), plus PNC   - Maternal and newborn mortality outcomes were mentioned but less so stillbirth. - Content mostly consistent across documents - ICs mostly focused on ANC and EMoNC; PADS attention mostly on EMoNC and quality of care - No document had content on respectful maternity care or family centred care | - Quality for MNH is an area of interest in the mindset of many of these investment cases - Absence of patient experience aspects of quality and an absence of clear linkage between health system investments and the improvements in health-area specific quality that are related - Mostly mentioned EMoNC, midwifery, referral, MPDSR - Barely mentioned patient experience or stillbirths | - Large proportion of strategies cover CHWs. - CH acknowledged as important in all ICs and PADS except Senegal - CH activities mostly promotional, preventive & curative - Some ICs/PADs highlight challenges in implementing CH activities & possible solutions - Payments & incentives: mostly mentioned in PADS and usually PBF | MAIN FINDING ACROSS CONTENT ANALYSES  **How are the themes described and framed in the documents?**  **CoC themes (MNH, AH)**  ***MNH***   - Included in all documents - Mostly focused on pregnancy and birth, but had critical areas for MNH missing   ***AH***   - Mostly included in highest burden adolescent pregnancy contexts; exception of some fragile settings that excluded - Services: various AH services, mostly SRH in ICs   **Systems themes (quality, community**)  **Quality**   - Varying quality definitions; lack of clarity - Different approaches reflected in documents – no standard quality of care approach - Quality core component (though variable approaches) – more focused on maternal interventions   **Community Health**   - Community a core strategy in all countries but 1 assessed but approach & definitions varied, including actors names - Community actors roles varied   The content analyses for QoC & CH show that there is no “one-size fits all” strategy/approach  **IC vs PAD: was there continuity across the studies for these themes?**  ***MNH*:** Consistent mostly; ICs strong on ANC and EMoNC; PADs also EMoNC and QoC  ***AH*:** Continuity for 2 countries. Otherwise, quite inconsistent, sometimes PAD had AH content not in IC; AH mentioned more in ICs than PADs  **Community**: Addressed but sometimes in a fragmented manner; PADs sometimes had non-IC issues |
| Measure | - Adolescent pregnancy more commonly tracked. Only 2 countries with multiple indicators - Despite planning, Cambodia PAD and Cote d’Ivoire had no indicators for AH - Senegal PAD had indicators, though none in IC | - ICs had comprehensive frameworks; PADs had fewer indicators for MNH (often SBA) - 5 PADS did not have PNC or small and sick newborn indicators - For outcomes, all ICs had maternal mortality reduction targets, 10 had newborn mortality targets. Newborn mortality was not targeted in any PAD | - Indicators are inconsistent; Quality indicators in PADs much more structural and less clinical / technical, focused on things like facility accreditation and availability of services | - No consistency in indicators across countries or between country documents, with the exception of Mali. - No CH objectives in any IC and only present in 3 PADs - Most PADs/ICs mention indicators to monitor CHW performance. Exception is Senegal (both), Cote d’Ivoire & Niger (ICs) | MAIN FINDING ACROSS CONTENT ANALYSES  How the thematic area is measured for the situation analysis and the results framework i.e. targets  **CoC themes (MNH, AH)**   - **MNH**: Indicators present +quality ones but the link not clear - **AH**: some areas were planned for but no indicators   **Systems themes (quality, community**)   - **CH**: Objectives mostly missing   **ICs vs PADs – what do we find across the documents**  ICs mostly looked at clinical aspects of quality, while PADs looked at health systems strengthening  **Overall findings:**   - MNH and AH had common indicators used (ANC, SBA) (adolescent pregnancy) - PADs mostly lacked newborn care & mortality indicators - **Community / Quality**: inconsistent indicators across countries and between country documents |
| Money | - Group 3 ICs allocated a percentage of budget to adolescents for particular adolescent programming items; Fewer country PADs in this group included allocations for AH. - PBF though was linked to specific activities - Despite planning, Cambodia PAD had no AH funding, while Senegal had in PAD not IC. Cote d’Ivoire had no AH money but had the content | - Differing approaches to funding descriptions prevented meaningful comparisons of MNH-specific budgets and allocations, though the allocations in the PADs are commensurate with the burden. - All ICs had some MNH content in budget - PADs had less, especially for PNC and newborn care | - The investments themselves are either limited to coverage indicators or focused on generic structural aspects of quality without linking to provision of care. | - Community health was budgeted for in the ICs of all countries except Guinea, and in all PADs except for Burkina Faso and Côte d'Ivoire. - PADs also discussed payments for CHWs, through PBF - A few mentioned non-financial incentives | The proposed and realised flow of funding around the thematic area of interest.  Were topics budgeted for?  **CoC themes (MNH, AH)**  **AH**: Not everything mentioned in the IC or PAD was always funded  **MNH**: Difficult to directly compare countries due to different funding approaches  **Systems themes (quality, community**)  CH: Majority of ICs and PADs had budget; Payments and incentives varied  **ICs vs PADs – what do we find across the documents**   - More MNH content in ICs than PADs, which had even less for newborn   **Overall**   - PBF and RBF used as a key funding mechanism especially in PADS, linked to activities |
